# Correction: Temporal proteomic profiling reveals insight into critical developmental processes and temperature-influenced physiological response differences in a bivalve mollusc

**DOI:** 10.1186/s12864-023-09145-3

**Published:** 2023-04-06

**Authors:** Shelly A. Wanamaker, Kaitlyn R. Mitchell, Rhonda Elliott Thompson, Benoit Eudeline, Brent Vadopalas, Emma B. Timmins-Schiffman, Steven B. Roberts

**Affiliations:** 1 School of Aquatic and Fishery Sciences, University of Washington, Seattle, Washington 98105 USA; 2 Taylor Shellfish Hatchery, Quilcene, Washington USA; 3 Washington Sea Grant, University of Washington, Seattle, Washington USA; 4 Department of Genome Sciences, University of Washington, Seattle, Washington USA

**Correction: BMC Genomics 21, 723 (2020)**

**https://doi.org/10.1186/s12864-020-07127-3**

Following publication of the original article [1], the first author would like to update her name.

The past author’s name is: Shelly A. Trigg

The current author’s name is: Shelly A. Wanamaker

The author group has been updated above, and the original article [1] has been corrected.
