# Supplementary material for: Temporal proteomic profiling reveals insight into critical developmental processes and temperature-influenced physiological response differences in a bivalve mollusc
Source: BMC Genomics. 2020 Oct 19;21:723. doi: 10.1186/s12864-020-07127-3 (PMC7574277; doi:10.1186/s12864-020-07127-3)
Supplement: Supplementary file 2 — Additional file 2 Supplemental_Figures. PCA of all technical replicate samples. Figure S1. PCA of all technical replicate samples. Figure S2. Preliminary principal component analysis plots of PC loadings for the top 100 ranked proteins. Figure S3. ANOVA-simultaneous component analysis plots of PC loadings for all proteins. [file 12864_2020_7127_MOESM2_ESM.pdf]

<u>Temporal proteomic profiling reveals insight into critical developmental processes and temperature-influenced physiological response differences in a bivalve mollusc</u>

Shelly A. Wanamaker ,[1]Kaitlyn R. Mitchell ,[1]Rhonda Elliott Thompson ,[1]Benoit Eudeline ,[2] Brent Vadopalas[3], Emma B. Timmins-Schiffman[4], and Steven B. Roberts[1]

[1]School of Aquatic and Fishery Sciences, University of Washington, Seattle, Washington, USA
[2]Taylor Shellfish Hatchery, Quilcene, Washington, USA
[3]Washington Sea Grant, University of Washington, Seattle, Washington, USA
[4]Department of Genome Sciences, University of Washington, Seattle, Washington, USA

Supplemental Figures:

Supplemental Figure 1. PCA of all technical replicate samples. (page 2)

Supplemental Figure 2. Principal component analysis plots of PC loadings for the top 100 ranked proteins. (page 2)

Supplemental Figure 3. ANOVA-simultaneous component analysis plots of PC loadings for all proteins. (page 3)

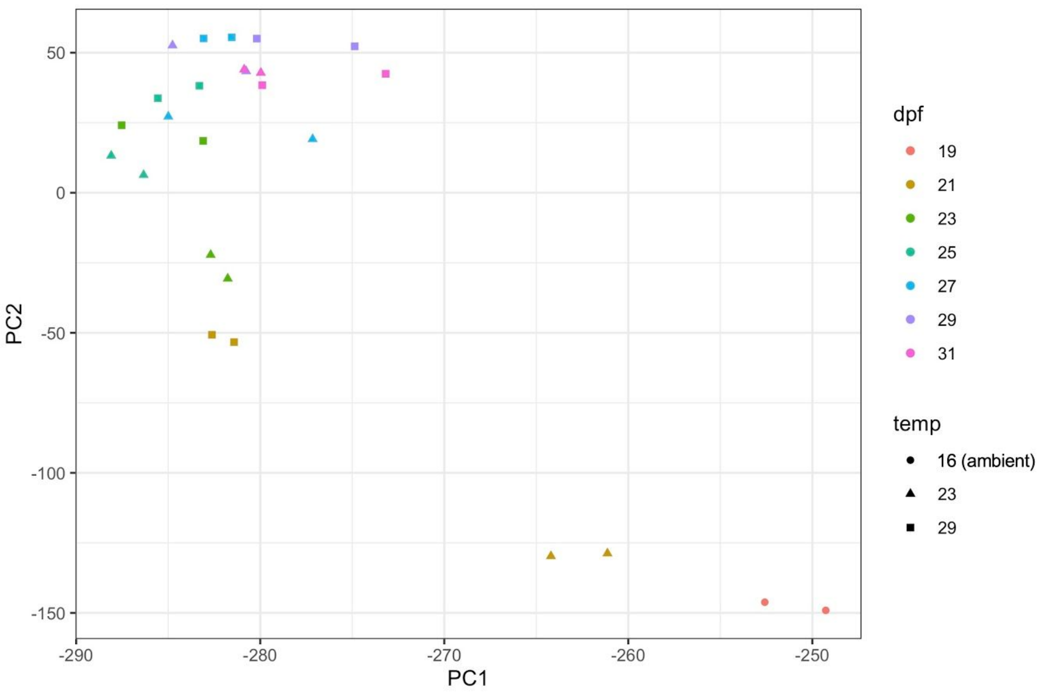

Supplemental Figure 1. PCA of all technical replicate samples.

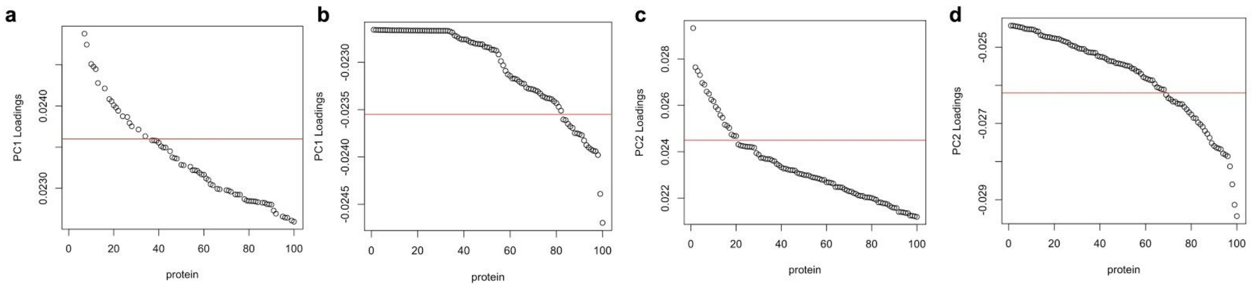

Supplemental Figure 2. Preliminary principal component analysis plots of PC loadings for the top 100 ranked proteins. (**a**) Proteins with the top 100 highest positive PC1 loadings thresholded (red line) at 0.0236. (**b**) Proteins with the top 100 lowest negative PC1 loadings thresholded (red line) at -0.02355. (**c**) Proteins with the top 100 highest positive PC2 loadings thresholded (red line) at 0.0245. (**d**) Proteins with the top 100 lowest negative PC2 loadings thresholded (red line) at -0.0262.

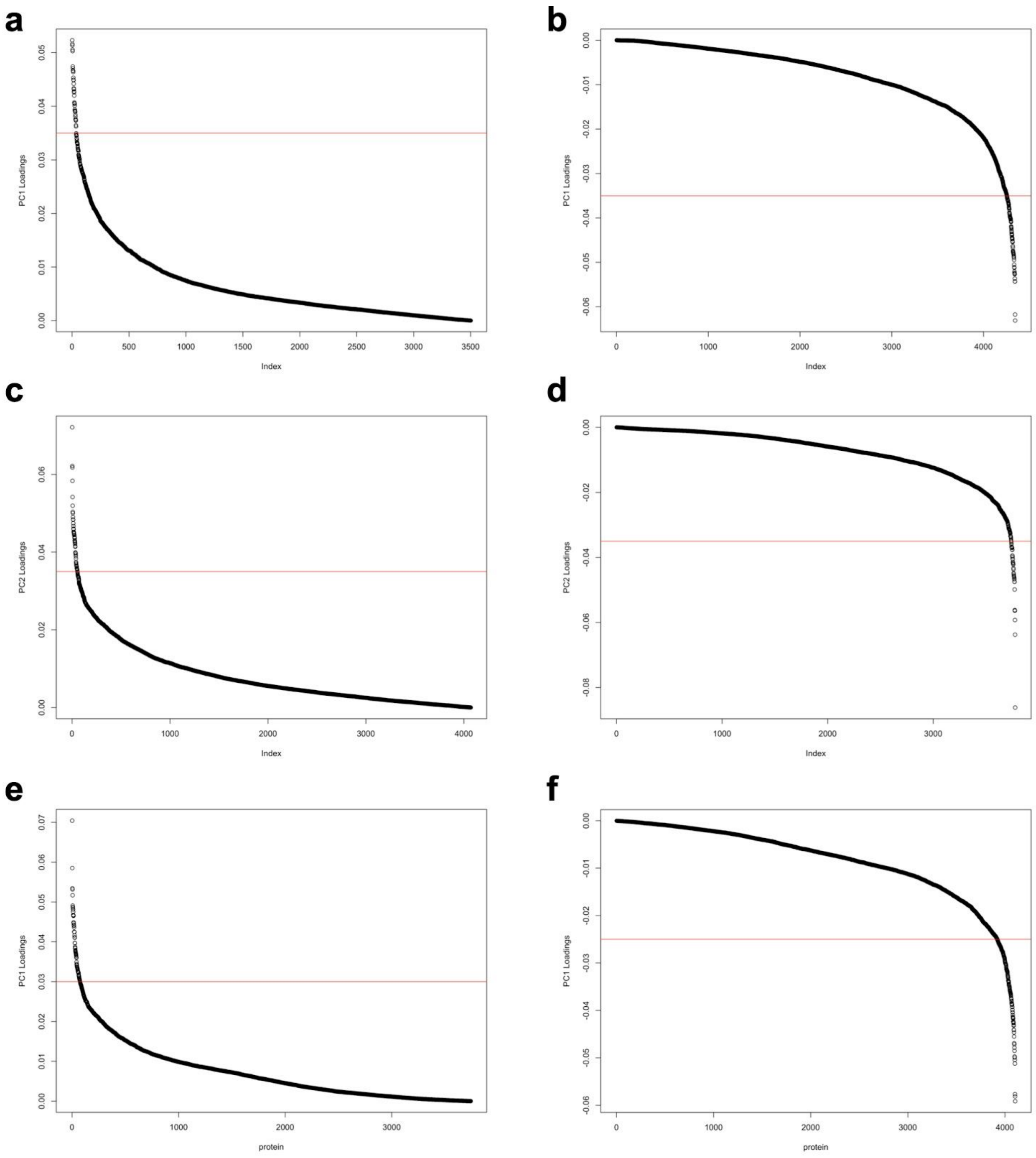

Supplemental Figure 3. ANOVA-simultaneous component analysis plots of PC loadings for all proteins. **(a)** Positive PC1 loadings for time effect. (**b**) Negative PC1 loadings for time effect. **(c)** Positive PC2 loadings for time effect. (**d**) Negative PC2 loadings for time effect. (**e**) Positive PC1 loadings for temperature effect. (**f**) Negative PC1 loadings for temperature effect. Red line indicates the significance threshold loadings value set at the point of diminishing returns (0.035, -0.035, 0.035, -0.035, 0.03, and -0.025, respectively).
